# Supplementary material for: The role of memory-dependent friction and solvent viscosity in isomerization kinetics in viscogenic media
Source: Nat Commun. 2024 May 4;15:3761. doi: 10.1038/s41467-024-48016-7 (PMC11069540; doi:10.1038/s41467-024-48016-7)
Supplement: Supplementary file 1 — Supplementary Information Document [file 41467_2024_48016_MOESM1_ESM.pdf]

The role of memory-dependent friction and solvent viscosity in isomerization kinetics in viscogenic media - Supplementary Information

Benjamin A. Dalton, Henrik Kiefer, and Roland R. Netz
Freie Universität Berlin, Fachbereich Physik, 14195 Berlin, Germany

Contents

Supplementary Note 1. Additional simulation details 2

Supplementary Note 2. Water-glycerol solvent viscosity 3

Supplementary Note 3. Position-dependent mass of the butane dihedral angle 5

Supplementary Note 4. The generalised Langevin equation (GLE) and memory kernel extraction 6

Supplementary Note 5. Comparison between models with rigid and flexible bond angles 8

Supplementary Note 6. Dihedral barrier recrossing 10

Supplementary Note 7. Translational diffusivity 12

Supplementary Note 8. Additional information for extended alkane and dipeptide simulations 13

Supplementary Note 9. Fitting the viscosity scaling for dihedral barrier-crossing times 15

Supplementary Note 10. Kramers' and Grote-Hynes predictions of butane and decane isomerization times 18

Supplementary Note 11. Butane dihedral memory kernels and fitting parameters 20

Supplementary Note 12. Effects of memory kernel oscillation on Grote-Hynes predictions 23

Supplementary Note 13. Water escape times as a solvent relaxation time scale 24

Supplementary Note 1. Additional simulation details

We conducted simulations of single n-alkane molecules and single amino acids in pure water and water-glycerol mixtures to investigate how changes in solvent viscosity affect their behavior. To manipulate the solvent viscosity, we either varied the mass of the water molecules or adjusted the concentration of the co-solvent glycerol. Simulation methods are described in the Methods section of the main manuscript. Here, we provide additional details regarding the analysis of simulation data. For all production runs, we save trajectories in blocks of 20 ns and analyse each block individually. We then compile the results for each block by averaging. For the mean first-passage times of butane, as shown in Fig. 2 in the main manuscript, we concatenate the 20 ns blocks into blocks of length 500 ns. For amino acid simulations, we exclude events where the ϕ-dihedral makes a full rotation or enters the rare $\phi = 55$ deg state, as these would require much longer simulations to generate converged trajectories. Any blocks that exhibit these rare events are discarded from the block averaging process. The butane dihedral is the angle subtended by the intersection of the $CH3_1$-$CH2_2$-$CH2_3$ and the $CH2_2$-$CH2_3$-$CH3_4$ planes. This angle is denoted as θ, where $\theta = 0$ degrees represents the trans-state. The dihedral undergoes transitions between the trans-state, and the two gauche-states, located at $\theta = \pm 120$ deg, by overcoming free energy barriers. The same conventions are used for all longer alkanes. Intramolecular interactions influence the free energy profile experienced by the dihedral, and these interactions are included explicitly in a dihedral potential. The large barrier separating the two gauche-states can be included via non-bonded 1-4 interactions or by explicitly incorporating the barrier into the dihedral potential function. To prevent gauche-to-gauche full rotations, we exclude non-bonded interactions and instead include a Ryckaert-Bellemans potential [1]. The Ryckaert-Bellemens potential is expressed as a fifth-order cosine power series: $V_{\mathrm{RB}}(\theta) = \sum_{n=0}^{5} v_n \cos^n(2\pi\theta/360)$, with standard coefficients $v_0 = 9.28$, $v_1 = 12.16$, $v_2 = -13.12$, $v_3 = -3.06$, $v_4 = 26.24$, and $v_5 = -31.50$, in units of kJmol^{-1}. In our simulations of alkanes, no gauche-to-gauche transitions were observed. For longer alkanes, we use the same dihedral potential with the same parameters for all dihedrals along the chain.

Supplementary Note 2. Water-glycerol solvent viscosity

For the water-glycerol mixtures, we evaluate the shear viscosity using the equilibrium Green-Kubo relation:

$$\eta_{\mathrm{GK}} = \frac{V}{10 k_{\mathrm{B}} T} \int_0^\infty \sum_{\alpha,\beta}^{3} \Big\langle \Pi_{\alpha\beta}(s) \Pi_{\alpha\beta}(s+\tau) \Big\rangle_s d\tau, \tag{S1}$$

where $\Pi_{\alpha\beta}$ is the trace-free stress tensor and $\langle \cdots \rangle_s$ is the ensemble average over s. The term inside the integral is the shear stress autocorrelation function (ACF), which we evaluate from MD simulations. In practice, Eq. S1 is a running integral with a finite upper time limit. For improved statistics, we average over N_{T} individual trajectories. For individual trajectories, the finite-time running integral approximation of Eq. S1 is:

$$I_j(t) = \frac{V}{10 k_{\mathrm{B}} T} \int_0^t \sum_{\alpha,\beta}^{3} \Big\langle \Pi_{\alpha\beta,j}(0) \Pi_{\alpha\beta,j}(\tau) \Big\rangle d\tau, \tag{S2}$$

where $j = 1, 2, ..., N_{\mathrm{T}}$. The average running integral is then:

$$I(t) = \frac{1}{N_{\mathrm{T}}} \sum_{j=1}^{N_{\mathrm{T}}} I_j(t). \tag{S3}$$

In Supplementary Figure 1A, we show the ensemble-averaged running integral $I(t)$ for pure water in atmospheric conditions with standard mass. We show the $I_j(t)$ from individual trajectories in blue with the result from Eq. S3 in black. On average, the stress ACFs decay after approximately 5 ps. However, the individual integrals are scattered. We select a plateau region, between the bounds of t_{min} and t_{max}, where Eq. S3 is well converged, and we approximate the solvent viscosity as the average of $I(t)$ in this region:

$$\eta = \frac{1}{t_{\mathrm{max}} - t_{\mathrm{min}}} \int_{t_{\mathrm{min}}}^{t_{\mathrm{max}}} I(t) dt. \tag{S4}$$

The red overlay in Supplementary Figure 1A indicates the selected plateau region. We perform this calculation for all water-glycerol solvent compositions. The numerical results are presented in Supplementary Table 1. In Supplementary Figure 1B, we show Eq. S3 for each water-glycerol composition, with plateau regions indicated in red. Likewise, the equivalent results for the super-heavy water are presented in Supplementary Figure 1C. We plot the results for Eq. S3 in Fig. 1E of the main text, where we show good agreement with an experimentally-derived empirical curve [2]. In Supplementary Figure 1D, we show the longest time scale τ_η for the average stress autocorrelation function, as given in Eq. S2. These time scales indicate the longest relaxation time in the internal shear stresses and hence the solvent stress relaxation.

Supplementary Table 1: Compositions of water-glycerol mixtures, indicating the number of water molecules N_w and the number of glycerol molecules N_{gly} for each mixture. The concentrations of glycerol are given as the mass percentages.

% glycerol	N_w	N_{gly}	η [mPa · s]	η/η_0
0	1500	0	0.86	1.0
10	1350	29	1.11	1.3
20	1200	59	1.54	1.8
30	1050	88	2.20	2.5
40	900	117	3.42	3.9
50	750	147	5.80	6.7
60	600	176	12.16	14.0

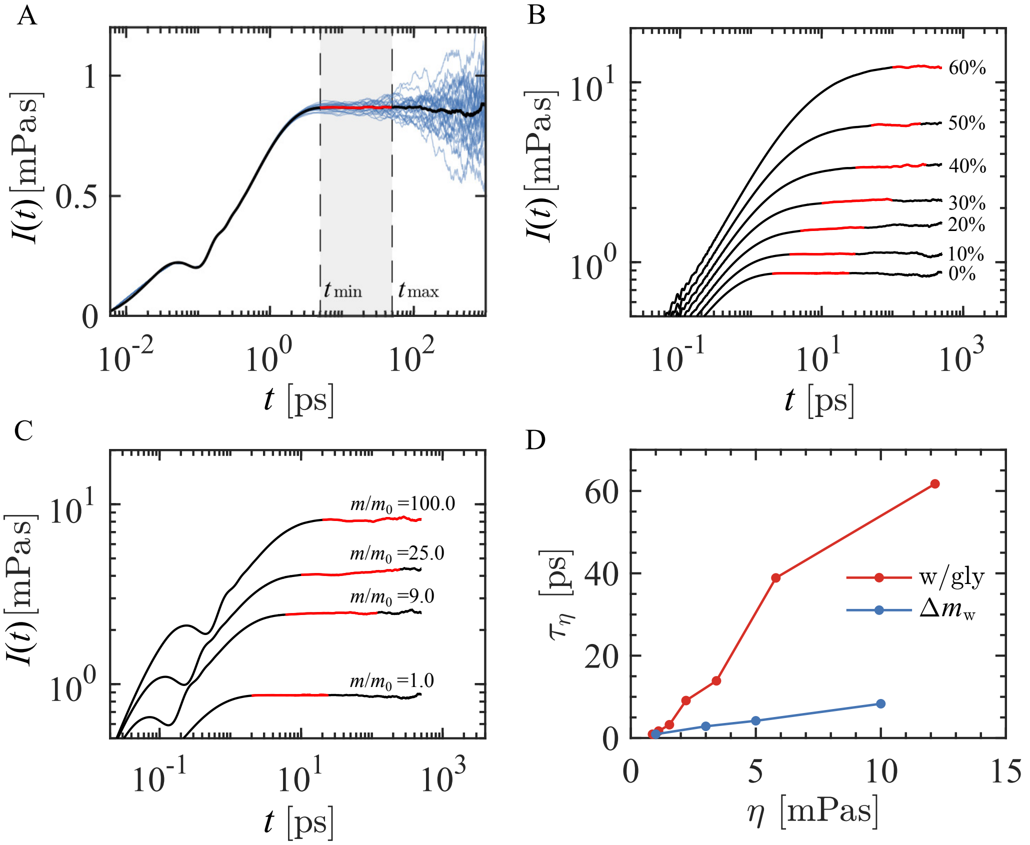

Supplementary Figure 1: Calculating viscosity for water-glycerol mixtures with the Green-Kubo method. (A) Running integral of stress ACF for pure water, according to Eq. S3. The black curve is the average of 25 trajectories. The individual trajectories are shown in blue. The plateau region (red overlay) is set by choosing the bounds $\mathrm{t_{min}}$ and $\mathrm{t_{max}}$. (B) The average running integrals for the range of glycerol concentrations, with the chosen plateau regions (shown in red). (C) The average running integrals for the super-heavy water solvent. (D) Longest time scale τ_η of the stress autocorrelation functions used to evaluate the running integrals for each system.

Supplementary Note 3. Position-dependent mass of the butane dihedral angle

In the main manuscript, we treat the effective mass of all dihedrals to be constant. This is an approximation. It is known that the rigid-angle butane model, introduced in Supplementary Note 5, exhibits position-dependent mass [3]. This is important when considering the position dependence of the friction memory kernel, which we neglect in this paper. Regardless, in Supplementary Figure 2, we show that the mass for the flexible-angle butane model used throughout this paper also depends on the position, which we calculate using histograms for the position-dependent dihedral velocity $\dot{\theta}(\theta)$, and hence the position-dependent equipartition theorem $m_{\mathrm{eff}}(\theta) = k_{\mathrm{B}}T/\langle\dot{\theta}(\theta)\rangle^2$. The values used for the constant mass are indicated in the figure. Note that in the main manuscript and all other sections of this SI, the effective, position-independent mass is simply denoted m.

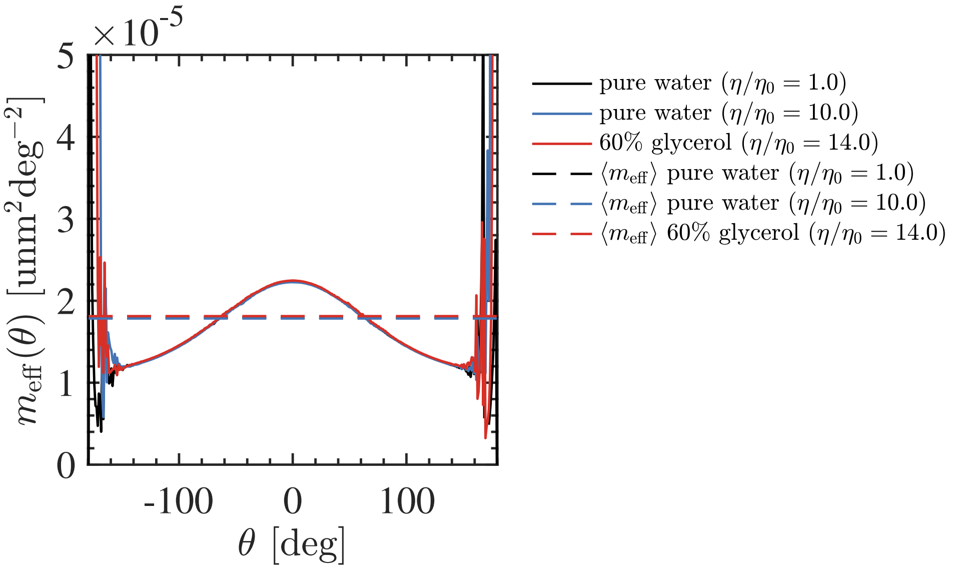

Supplementary Figure 2: Effective mass profiles for the butane dihedral, evaluated over a range of solvent viscosity conditions. The profiles are evaluated using a position-dependent equipartition theorem $m_{\mathrm{eff}}(\theta) = k_{\mathrm{B}}T/\langle\dot{\theta}(\theta)\rangle^2$. The dashed lines show the mean values that are position independent, which are used throughout the main manuscript.

Supplementary Note 4. The generalised Langevin equation (GLE) and memory kernel extraction

MD simulation trajectories are projected onto a either a dihedral-angle reaction coordinate $\theta(t)$, or an intra-atomic distance reaction coordinate $d_{14}(t)$. The dynamics of these reaction coordinates are then mapped onto the 1D GLE:

$$m\ddot{q}(t) = -\int_0^t \Gamma(t-t')\dot{q}(t')dt' - \frac{\partial}{\partial q}U\big[q(t)\big] + F_R(t), \tag{S5}$$

where q is any general reaction coordinate. Here, we exclude any position dependence of the memory kernel [3]. $F_R(t)$ is the random force term, which has a zero mean $\langle F_R(t)\rangle = 0$, and satisfies the fluctuation-dissipation theorem $\langle F_R(t)F_R(t')\rangle = k_\mathrm{B}T\Gamma(|t-t'|)$. $U(q)$ is the potential of mean force, which is extracted uniquely for each system according to $U(q) = -k_\mathrm{B}T\log\big[\rho(q)\big]$, where $\rho(q)$ is the probability density over $q(t)$. The total friction on the dihedral is given by the converged plateau value of the integrated memory kernel:

$$\gamma = G(t\to\infty) = \int_0^\infty \Gamma(t)dt, \tag{S6}$$

where $G(t) = \int_0^t \Gamma(t')dt'$ is the running integral of the memory kernel.

To extract $\Gamma(t)$, we use the running integral extraction scheme in the presence of general potentials [4, 5]. We correlate Eq. S5 with the initial position of the dihedral angle $q(0)$:

$$m\Big\langle q(0)\ddot{q}(t)\Big\rangle = -\int_0^t \Gamma(t-t')\Big\langle q(0)\dot{q}(t')dt'\Big\rangle - \Big\langle q(0)\nabla U\big[q(t)\big]\Big\rangle + \Big\langle q(0)F_R(t)\Big\rangle, \tag{S7}$$

and with the initial velocity of the dihedral angle $\dot{q}(0)$:

$$m\Big\langle \dot{q}(0)\ddot{q}(t)\Big\rangle = -\int_0^t \Gamma(t-t')\Big\langle \dot{q}(0)\dot{q}(t')dt'\Big\rangle - \Big\langle \dot{q}(0)\nabla U\big[q(t)\big]\Big\rangle + \Big\langle \dot{q}(0)F_R(t)\Big\rangle, \tag{S8}$$

Due to the original orthogonality relations that hold for the GLE in Eq. S5, both $\big\langle q(0)F_R(t)\big\rangle = 0$ and $\big\langle \dot{q}(0)F_R(t)\big\rangle = 0$. We write Eqs. S7 and S8 in terms of the position-velocity and velocity-velocity correlation functions $C^{q\dot{q}}(t) = \big\langle q(0)\dot{q}(t)\big\rangle$ and $C^{\dot{q}\dot{q}}(t) = \big\langle \dot{q}(0)\dot{q}(t)\big\rangle$, respectively, as well as the correlations between the dihedral angle and the PMF gradients $C^{q\nabla U}(t) = \big\langle q(0)\nabla U\big[q(t)\big]\big\rangle$, and the velocity of the dihedral angle and the PMF gradients $C^{q\nabla U}(t) = \big\langle \dot{q}(0)\nabla U\big[q(t)\big]\big\rangle$:

$$m\frac{d}{dt}C^{q\dot{q}}(t) = -\int_0^t \Gamma(t')C^{q\dot{q}}(t-t')dt' - C^{q\nabla U}(t), \tag{S9}$$

$$m\frac{d}{dt}C^{\dot{q}\dot{q}}(t) = -\int_0^t \Gamma(t')C^{\dot{q}\dot{q}}(t-t')dt' - C^{\dot{q}\nabla U}(t). \tag{S10}$$

We integrate Eq. S10 in the time domain and obtain an equation in terms of $G(t) = \int_0^t \Gamma(t')dt'$:

$$mC^{\dot{q}\dot{q}}(t) - mC^{\dot{q}\dot{q}}(0) = -\int_0^t G(t-t')C^{\dot{q}\dot{q}}(t'')dt'' + C^{q\nabla U}(t) - C^{q\nabla U}(0). \tag{S11}$$

Using the identity $\frac{d}{dt}C^{q\dot{q}}(t) = C^{q\ddot{q}}(t) = -C^{\dot{q}\dot{q}}(t)$, we evaluate Eq. S9 at t=0, and obtain $mC^{\dot{q}\dot{q}}(0) = C^{q\nabla U}(0)$. It follows that:

$$\frac{C^{\dot{q}\dot{q}}(t)}{C^{\dot{q}\dot{q}}(0)}C^{q\nabla U}(0) = C^{q\nabla U}(t) - \int_0^t G(t-t')C^{\dot{q}\dot{q}}(t')dt'. \tag{S12}$$

Eq. S12 can be discretized using the trapezoidal rule for numerical integration. Using $G(0) = 0$, we arrive at the following numerical extraction scheme, which we use to extract the discrete representation of $G(t)$ directly from the

trajectory of a given dihedral angle:

$$G_i = \begin{cases} 0, & i=0 \\ \frac{2}{\Delta t C_0^{\dot{q}\dot{q}}}\left[C_1^{\nabla U q} - \frac{C_0^{\nabla U q}}{C_0^{\dot{q}\dot{q}}} C_1^{\dot{q}\dot{q}}\right], & i=1 \\ \frac{2}{\Delta t C_0^{\dot{q}\dot{q}}}\left[C_i^{\nabla U q} - \frac{C_0^{\nabla U q}}{C_0^{\dot{q}\dot{q}}} C_i^{\dot{q}\dot{q}} - \Delta t \sum_{j=1}^{i-1} G_j C_{i-j}^{\dot{q}\dot{q}}\right], & i>1 \end{cases} \tag{S13}$$

where $C_i^{\dot{q}\dot{q}}$ and $C_i^{\nabla U q}$ are the discretized representations of the velocity-velocity correlation function and the correlations between the dihedral angle and the gradients of the PMF, respectively.

Supplementary Note 5. Comparison between models with rigid and flexible bond angles

It is known that the angles of the bonds that form the dihedral play a significant role in the reaction dynamics of the dihedral and can significantly alter the transition barrier. Here, we compare results from the butane model used in the main manuscript, which has flexible bond angles, to two alternative models, both of which have rigidly constrained bond angles.

In Supplementary Figure 3A, we show normalized memory kernels for the butane dihedral angle with flexible bond angles (same as Fig. 2B of the main manuscript) and for butane with rigidly-constrained bond angles (constrained using the SHAKE algorithm [6]), simulated in neat water. Both models exclude non-bonded interactions between the 1-4 CH groups. For both systems, the dihedral free energy profile is fully accounted for by a Ryckaert-Bellemen potential, as discussed in Supplementary Note 1. These are the Flexible (RB) and Rigid (RB) models presented in Supplementary Figure 3. The aim is to show the contributions to the memory kernel due to the inclusion of oscillating bond-angle degrees of freedom, which are orthogonal to the dihedral angle. Additionally, we also include the memory kernel for dihedral dynamics regenerated using the rigid model used by Daldrop et. al [7], which has rigidly constrained bond angles and includes non-bonded interactions between the 1-4 CH groups. The inclusion of non-bonded 1-4 interactions prevents gauche-to-gauche transitions. To include isomerization barriers, a periodic potential with of the form $A(1+\cos(2\pi n/360))$, where $n = 3$ and $A = 5.9$ kJmol^{-1}, is used in place of a Ryckaert-Bellemen dihedral potential.

In Supplementary Figure 3B-C, we show the mean first-passage times (τ_{MFP}) and total friction (γ) for the flexible-angle butane model with Ryckaert-Bellemen dihedral potential (which is the same as presented in the main manuscript) and for the rigid Daldrop model. In Supplementary Figure 3C, where we compare the total friction on the dihedral, we also explicitly show the results from [7], where the low viscosity regime ($\eta/\eta_0 = 0.1$ and 0.3) was also investigated. For $\eta/\eta_0 \geq 1.0$, all models exhibit sub-linear scaling. We include a $(\eta/\eta_0)^{0.4}$ scaling line as a visual guide for the approximate behaviour of the friction over this range of viscosity.

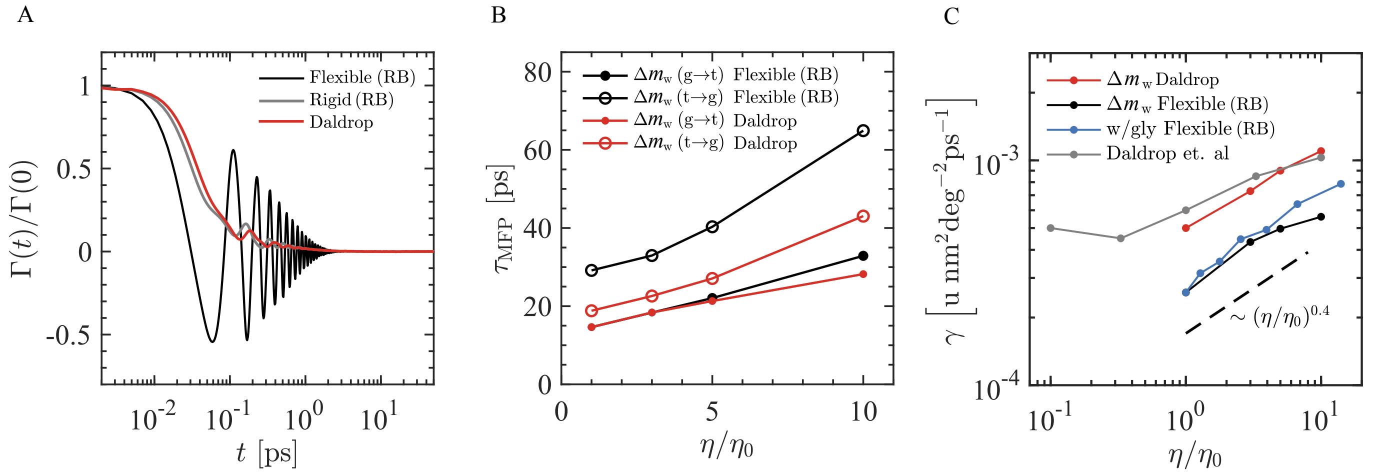

Supplementary Figure 3: Comparison of memory kernels and barrier crossing times for butane molecules with flexible and rigidly constrained bond angles. (A) Memory kernels for butane simulated in pure water with standard water mass ($\eta/\eta_0 = 1$). The Flexible (RB) and Rigid (RB) models have a Ryckaert-Bellemen dihedral potential and excluded non-bonded 1-4 interactions. The rigid butane is constrained using the SHAKE algorithm [6]. While the memory kernel for the rigid model exhibits weak oscillating modes, the flexible bond molecule has strongly oscillating memory effects in the dihedral dynamics. Note that both models have rigidly constrained bond lengths. For all data in the main text we use the flexible bond-angle model. We also include the memory kernel for a system generated using the rigid-angle model of Daldrop et al. [7]. (B) Barrier crossing times for butane super-heavy water, plotted as a function of viscosity. The black data points are the same as those shown in Fig. 2 of the main manuscript for the super-heavy water systems (flexible bond angles). Red data points are for the rigid-angle Daldrop model. (C) Comparison of total friction γ for the flexible-angle and Daldrop models. Results presented by Daldrop et. al [7] are also explicitly shown, which includes results for $\eta/\eta_0 < 1$. A $(\eta/\eta_0)^{0.4}$ scaling line is shown as a guide.

In Supplementary Figure 4, we compare the conformational probability densities for a butane model with flexible bond angles, and a model with rigid bond angles, to the gas phase result. In the gas phases, it is assumed that only the potential acting on the dihedral influences the conformational densities. As described in Supplementary Note 1, we use a Ryckaert-Bellemen dihedral potential [1] $V_{\mathrm{RB}}(\theta)$. The associated gas-phase dihedral probability density will be $P_{\mathrm{RB}}(\theta) = \exp[-V_{\mathrm{RB}}(\theta)/k_{\mathrm{B}}T]/\int_{-180}^{180} d\theta \exp[-V_{\mathrm{RB}}(\theta)/k_{\mathrm{B}}T]$. In Supplementary Figure 4, we show that solvation

effects for the rigid model are significant. However, the effects are only small for the flexible model. We note that Rosenberg et al. used a model with flexible bond lengths and angles in their original work studying the effects of solvent material properties on dihedral isomerization [8]. However, studies of butane that focus on the solvation effects on conformational equilibrium typically use a model that has rigid bond lengths and angles [9–11].

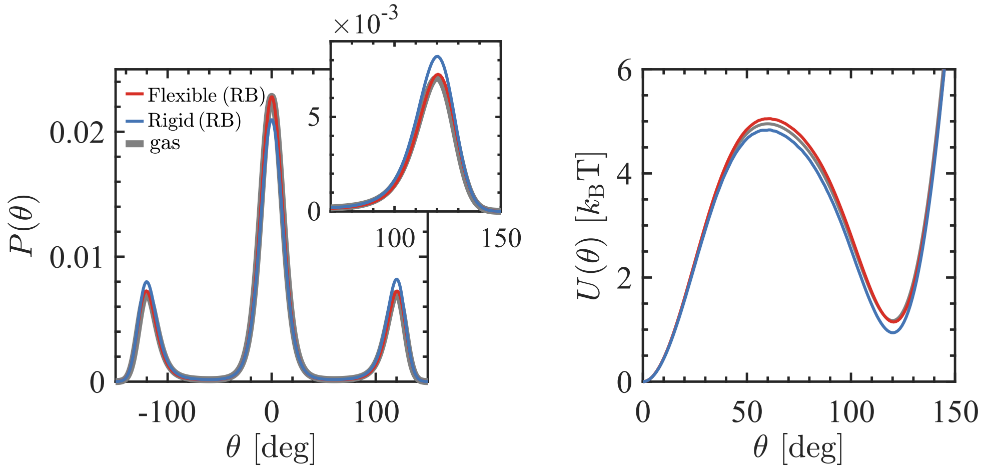

Supplementary Figure 4: Comparison of conformation equilibrium for various butane models. Dihedral angle probabilities densities (left) and free energy profiles (right) for gas phase butane (grey), butane with flexible bond angles in neat water (red), and butane with rigid bond angles in neat water (blue). The gas phase profiles are determined directly from the Ryckaert-Bellemen potential imposed on the dihedral degree of freedom, such that in the gas phase $U(\theta) = V_{\mathrm{RB}}(\theta)$ ($T = 300$ K).

Supplementary Note 6. Dihedral barrier recrossing

Butane exhibits significant state-recrossing dynamics, where upon entering a new state, the dihedral can either quickly move to a neighbouring state or remain in the current state briefly before transitioning again [12]. This behaviour is typically attributed to correlated barrier-crossing [13]. We note that this behaviour is distinct from barrier recrossing, where a system makes multiple crossings of a transition state during a single excursion between reactant and product states. This later class of recrossing is known to be common in protein folding, for example. However, such recrossing does not occur in the trajectories analysed in the present manuscript. In the following discussion, we only consider state recrossing, which we simply refer to as recrossing.

In Supplementary Figure 5A, we show a short trajectory segment for a butane dihedral in super-heavy water with $\eta/\eta_0 = 10.0$, which displays multiple instances of immediate state recrossing and multiple brief state residencies. In Supplementary Figure 5B, we show various definitions of barrier crossing times. The aim here is to consider the distribution and mean for each definition, as we recently discussed [12]. τ^i_{AFP} indicates a sequence of all-to-first passage (AFP) times, where i is an event index. The all-to-first passage times account for each crossing of the trans-state before crossing into the gauche-state. The time interval between consecutive crossings within a single state and the first crossing into the subsequent state are the events that contribute to the all-to-first passage time distribution. The mean all-to-first passage time is $\tau_{\mathrm{AFP}} = \langle \tau^i_{\mathrm{AFP}} \rangle_i$, where $\langle ... \rangle_i$ is the ensemble average over index i. The single τ^i_{FFP}, shown in Supplementary Figure 5B, is an example of a first-to-first passage (FFP) event, defined as the first crossing into a new state while disregarding any subsequent crossings. The complete series of τ^i_{FFP}s obtained throughout a trajectory is used to calculate the mean first-first passage time τ_{FFP}, which is equivalent to the mean waiting time, which is often used as a measure of a reaction time. To compile a distribution of crossing times that neglects recrossing events, we introduce a delay time δt. When entering a new state, any crossings occurring during the initial period are discarded until a chosen delay time, represented by δt, has passed. Once the delay time has elapsed, we compile all subsequent crossing times τ^i_{DAFP} into a distribution and hence compile a delayed all-first passage (DAFP) time distribution, and a mean delayed all-first passage time τ_{DAFP}.

In Supplementary Figure 5C, we show the distributions for the all-to-first, first-to-first, and delayed all-to-first passage times, and we compare butane in neat water to butane in super-heavy water with $m/m_0 = 100$. Recrossing manifests as additional fast components in the barrier-crossing time distributions. To emphasize the deviations from a single-component exponentially distributed process, we fit single-component exponentials to the long-time tail of each distribution. The deviations are amplified in the first-to-first distributions. However, they are eliminated in the delayed all-to-first distributions. By introducing the delay time δt, we have filtered the memory-induced fast correlated-recrossing modes from the distribution, while maintaining the time scale of the slowest exponential mode. Intuitively, this is equivalent to preparing an ensemble of systems in a state such that the system has no memory of previous transition events, and then calculating the mean first-passage times from that state. In Supplementary Figure 5D, we show that the choice of distribution has a dramatic effect on the mean barrier-crossing times. For the super-heavy water system, τ_{FFP} is independent of viscosity. τ_{AFP} and τ_{DAFP} are both dependent on viscosity. However, due to the presence of fast, multi-exponential components in the τ^i_{AFP} distributions, especially for higher viscosities, τ_{AFP} deviates from τ_{DAFP}. In the main manuscript, we present the mean delayed all-to-first passage times to emphasize the coupling between the viscosity and the independent, or uncorrelated, contribution to the barrier-crossing process. For all systems in this study, we use a delay time of $\delta t = 25$ ps.

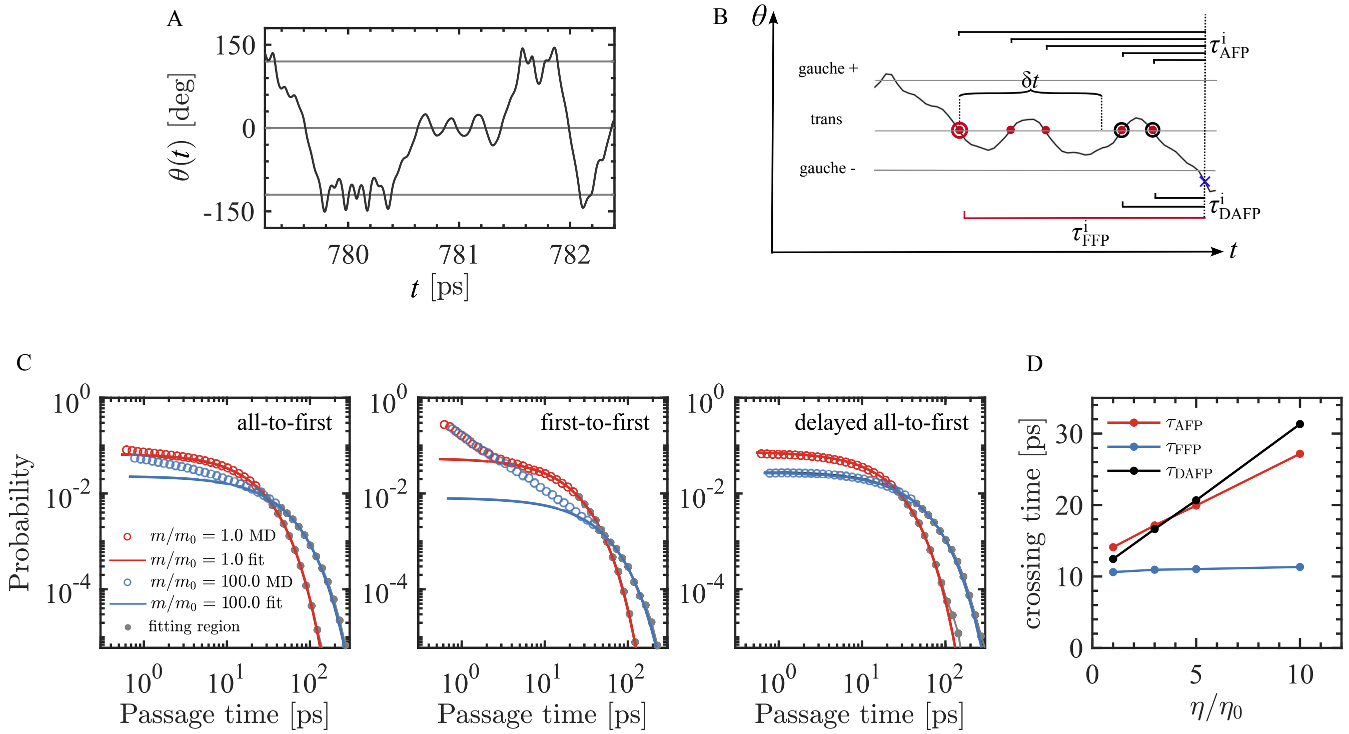

Supplementary Figure 5: (A) A short trajectory segment showing a combination of recrossing events and short residencies. (B) An example trajectory segment showing the butane dihedral as it passes through 3 isomeric states. The red points show dihedral crossings over the trans-state minimum. The open red circle shows the first crossing into the trans-state minimum, and the blue cross shows the first crossing to the subsequent gauche-state minimum. The black circles show the crossings of the trans-state minimum that occur later than δt. The black lines at the top of the figure show the all-to-first passage times connecting each crossing of the trans-state minimum to the first arrival in the gauche-state. The red line at the bottom shows the transition time for the first-to-first passage. The two black lines at the bottom show all of the passage times from the trans to the gauche-state after δt for this example transition. These are the delayed-all-to-first events. (C) Distributions of the all-to-first, first-to-first, and delayed all-to-first passage times, taken over a long butane trajectory (total time 2 μs) for trans-to-gauche transitions. Distributions are accumulated using exponentially increasing bin widths, suitable for plotting on a logarithmic scale. Single-component exponentials are fit to the long-time tails for each distribution. The region of the fitting is indicated with grey points. (D) The viscosity dependence for the mean first-passage times, calculated for the all-to-first, first-to-first, and delayed all-to-first passage time distributions.

Supplementary Note 7. Translational diffusivity

The translational diffusion coefficients for the butane centre of mass, as shown in Fig. 2E of the main text, are determined by fitting the long-time diffusive regime for the centre of mass mean squared displacements (MSD). We show the MSDs for all solvent conditions in Supplementary Figure 6A, and we indicate the long-time diffusive regimes in red, which are approximately linear (compare to the linear scaling lines on each figure). The MSDs are evaluated using the *gmx msd* GROMACS library. The diffusion coefficient is given by the gradient of a linear fit, which we rescale using D_0, i.e. the diffusion coefficient for the 0% glycerol, or m/m = 1, in the main manuscript.

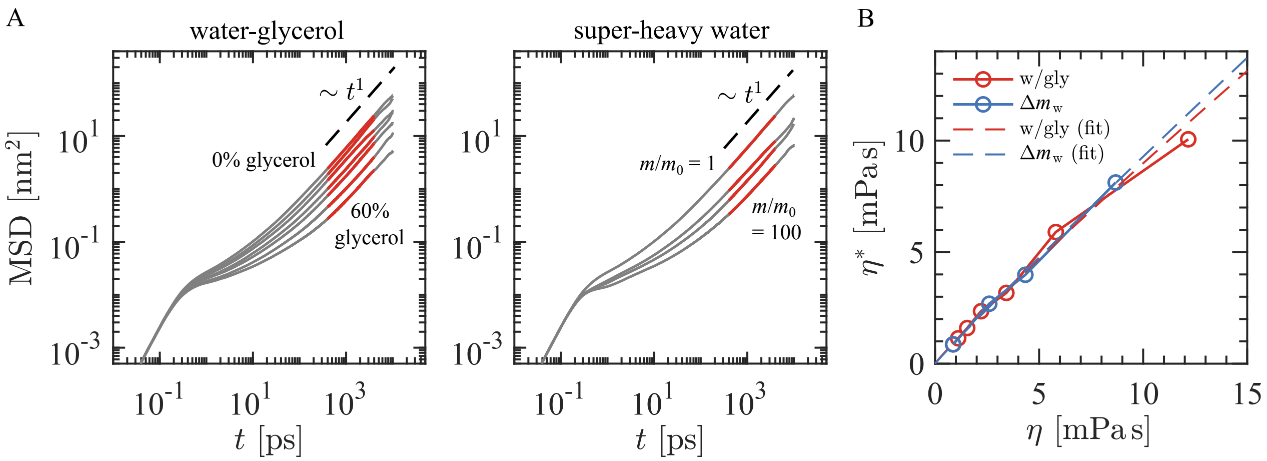

Supplementary Figure 6: (A) Mean squared displacements (MSD) for the butane centre of mass translational diffusion. For the water-glycerol system, the 0% glycerol and 60% glycerol curves are indicated. The MSDs for the remaining concentrations are organised monotonically between these two extremes. We also indicate m/m = 1 and 100 for the low and high viscosity curves for the super-heavy water. The long-time diffusive regimes are indicated in red for each MSD. The black dashed lines show linear scaling. (B) A local viscosity η^* in an inhomogeneous medium, as determined using the centre of mass transitional diffusion of the butane molecule (Eq. S14) under the various solvent conditions. Fits are given by $\eta^* = \alpha\eta^\beta$.

The translational diffusion coefficients evaluated in Supplementary Figure 6A can be used to quantify whether the butane translational motion experiences a reduced local viscosity compared to the bulk viscosity. Such a local reduction has been discussed for the diffusion of probe molecules in inhomogeneous media when the probe particle is small compared to some inhomogeneous length scale [14, 15]. Such a reduced local viscosity (η^*) can be quantified by

$$\eta^* = \eta_0 \frac{D_0^{\mathrm{tr}}}{D^{\mathrm{tr}}}. \tag{S14}$$

Here, η_0 and D_0^{tr} are the viscosity and translational diffusion coefficients in neat water. Results for η^* are shown in Supplementary Figure 6B, calculated using the butane center of mass to represent the probe particle. Using the relationship $\eta^* \sim \eta^\beta$, we say that influences from inhomogeneous viscosity are present when $\beta < 1$, and that when $\beta = 1$, the translation diffusion of the particle is determined by the bulk viscosity. In Supplementary Figure 6B, we show fitting results for $\eta^* = \alpha\eta^\beta$. The fits yield $\beta = 0.96$ and 0.93 for the super-heavy water and water-glycerol mixtures, respectively, suggesting that there is a negligible deviation from macroscopic viscosity. In the inset of Fig. 2E in the main manuscript, we show that $\eta/\eta_0 \approx D_0^{\mathrm{tr}}/D^{\mathrm{tr}}$, with an onset of deviations appearing at 60% glycerol mixtures.

Supplementary Note 8. Additional information for extended alkane and dipeptide simulations

For all alkane chains, we exclude non-bonded 1-4 Lennard-Jones interactions and hence include the Ryckaert-Bellemen dihedral potentials. In Supplementary Figure 7A, we show the free energy profiles, extracted from MD simulations, for the inner-most and outer-most dihedrals (see Fig. 3A from the main manuscript and Supplementary Figure 8A). We see that all free energy profiles are similar, with small deviations. In Supplementary Figure 7B, we show the relative changes in the barrier-crossing times for the inner dihedrals in neat water, plotted as a function of backbone length, as well as the corresponding relative changes in the barrier heights. Here, we use the butane times and heights as the reference values. Overall, we see that there are slight variations in the barrier heights, and the sensitivities of the gauche→trans and trans→gauche transitions are similar, with slight differences that are consistent with the trends observed for the barrier-height dependencies.

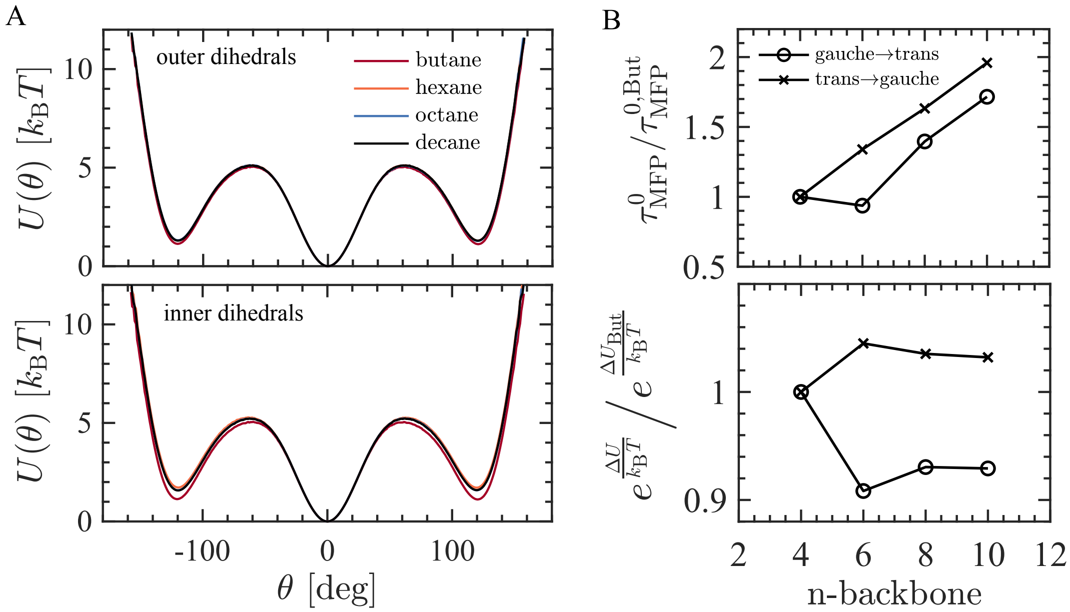

Supplementary Figure 7: (A) Free energy profiles for the outer and inner dihedrals of various alkane chains. All dihedral potentials are included using Ryckaert-Bellemen potentials. (B) Barrier crossing times in neat water τ_0, plotted as a function of n-alkane backbone length (in numbers of carbon atoms), for the inner-most dihedral of each n-alkane chain. Absolute values are shown in Fig. 3D of the main manuscript. To observe the relative sensitivity, we rescale by the reaction times for butane $\tau^{0,\text{But}}_{\text{MFP}}$. Therefore, we can observe the relative sensitivity for the two different transitions (gauche-trans and trans-gauche) as a function of backbone length. Relative changes in free energy barrier heights (see (A)), plotted as a function of n-alkane backbone length. ΔU is the barrier height in either state and ΔU_{But} is the corresponding height for butane. We calculate the Boltzmann factor associated with each transition and rescale by the butane value.

Additionally, we show the viscosity-dependence of the τ_{MFP} scaling along the sequence of dihedrals in the decane molecule. The chain containing 7 dihedrals is symmetric around the 4th dihedral (Supplementary Figure 8A.) In Supplementary Figure 8B, we can see that the different dihedrals exhibit slightly different barrier-crossing scaling. Regardless, the scaling for the two different solvent types diverges for all dihedrals between $\eta/\eta_0 = 3$ and 5.

In the inset of Fig. 3C in the main manuscript, we see that the phenylalanine barrier height ($\Delta U = 2.3\ k_{\text{B}}$T) is 20% higher than the alanine barrier ($\Delta U = 1.8\ k_{\text{B}}$T). To approximate whether the increase of τ^0_{MFP} is due to the change in barrier height, we use a simple Arrhenius model assuming that changes in the relevant curvatures of the free energy profile are negligible. For alanine in standard water $\tau^0_{\text{MFP}} = A\text{e}^{1.8} = 11.7$ ps, such that $A = 1.93$ ps. If the increase in τ^0_{MFP} is entirely due to changes in U, then for phenylalanine, we should expect $\tau^0_{\text{MFP}} = 1.93\text{e}^{2.3} = 19.1$ ps, which is 40% below the measured value. We suggest, then, that the difference is due to the influence of the large benzyl side group that distinguishes the phenylalanine and the alanine molecules.

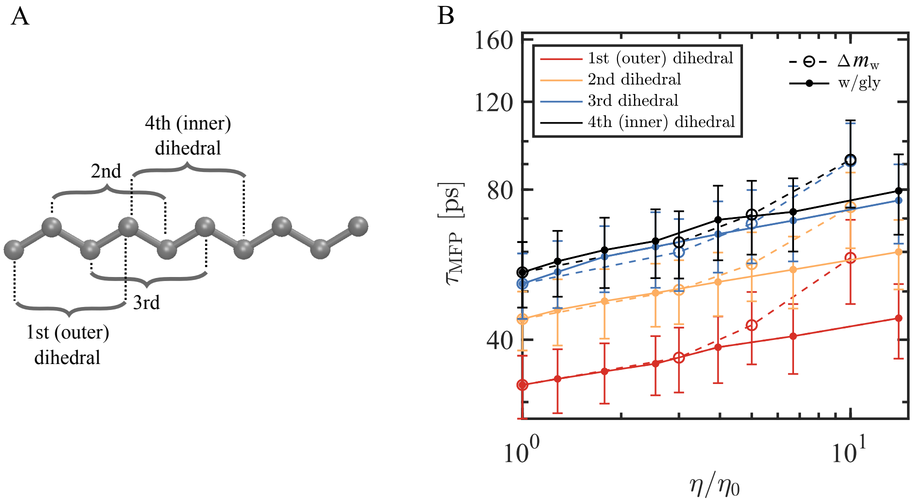

Supplementary Figure 8: Viscosity scaling of the decane dihedral barrier-crossing times. (A) Location of dihedrals. The chain is symmetric around the 4th (inner) dihedral. Only the 1st, 2nd, 3rd, and 4th locations are indicated. (B) Barrier crossing times as a function of viscosity for the set of dihedrals in the two different solvent types. Error bars are standard deviations calculated over 25 simulations at 20 ns length each.

Supplementary Note 9. Fitting the viscosity scaling for dihedral barrier-crossing times

The viscosity scaling of the barrier-crossing times for all molecules is fitted to a power law: $\tau_{\mathrm{MFP}}(\eta/\eta_0) = \alpha(\eta/\eta_0)^\beta + \varepsilon$. The results for α, β, and ε, as used in Fig. 3E of the main manuscript, are obtained by fits to the τ_{MFP} values, as measured in the simulation. We include two constraints on the fitting: $\beta \leq 1$ and $\varepsilon \geq 0$. In Supplementary Figure 9, we show the $\tau_{\mathrm{MFP}}(\eta/\eta_0)$ for the inner-most dihedral over all alkanes, and the fits. In Supplementary Figure 10, we show the viscosity scaling for the outer-most dihedral. The viscosity scaling and subsequent fitting for the alanine and phenylalanine are also shown (Supplementary Figure 11). Note that for the alkane inner dihedrals, for the case of butane and hexane in the super-heavy water solvent (Figs. Supplementary Figure 9A and B), we only fit $\eta/\eta_0 = 3, 5, 10$. This scaling is known to be linear in the high-viscosity regimes [7], but exhibit deviations from linearity in the low viscosity regime. We neglect the first data point $\eta/\eta_0 = 1$ to avoid including the non-linear regime, for which we would obtain $\beta > 1$.

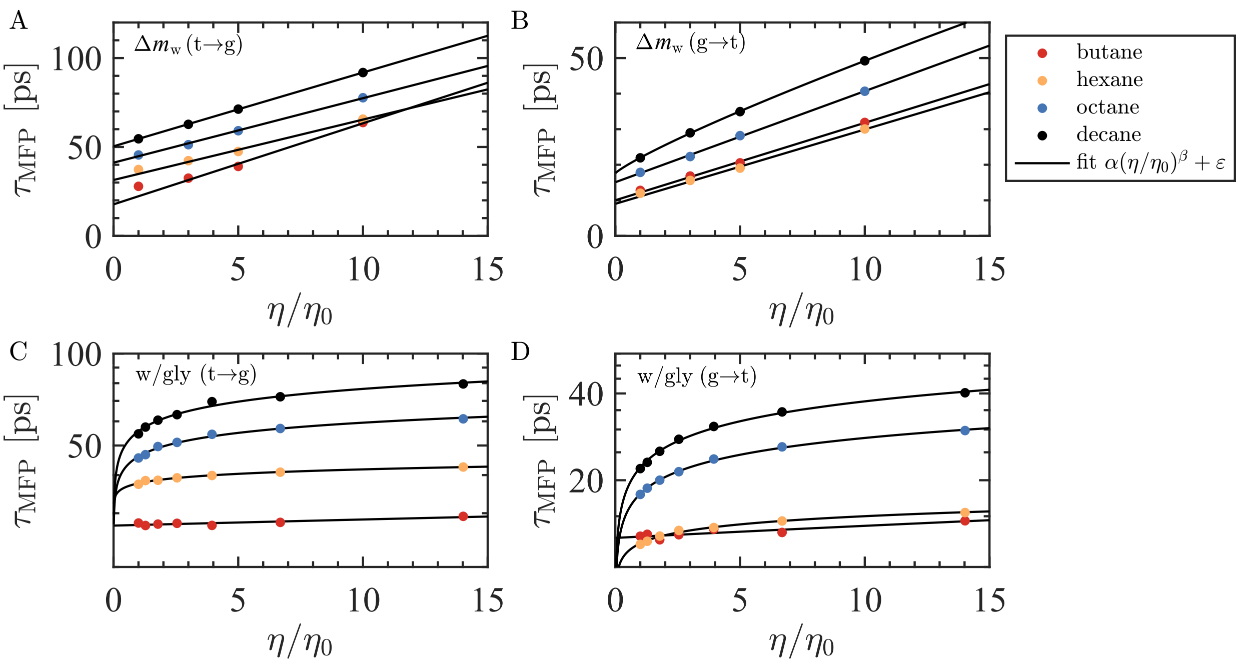

Supplementary Figure 9: Viscosity scaling of τ_{MFP} for the inner-most dihedral of the four alkanes. Coloured points are simulation data. The black lines are fits to $\tau_{\mathrm{MFP}}(\eta/\eta_0) = \alpha(\eta/\eta_0)^\beta + \varepsilon$. (A) and (B) Super-heavy water trans→gauche and gauche→trans transitions, respectively. (C) and (D) Mixed water and glycerol solvent trans→gauche and gauche→trans transitions, respectively.

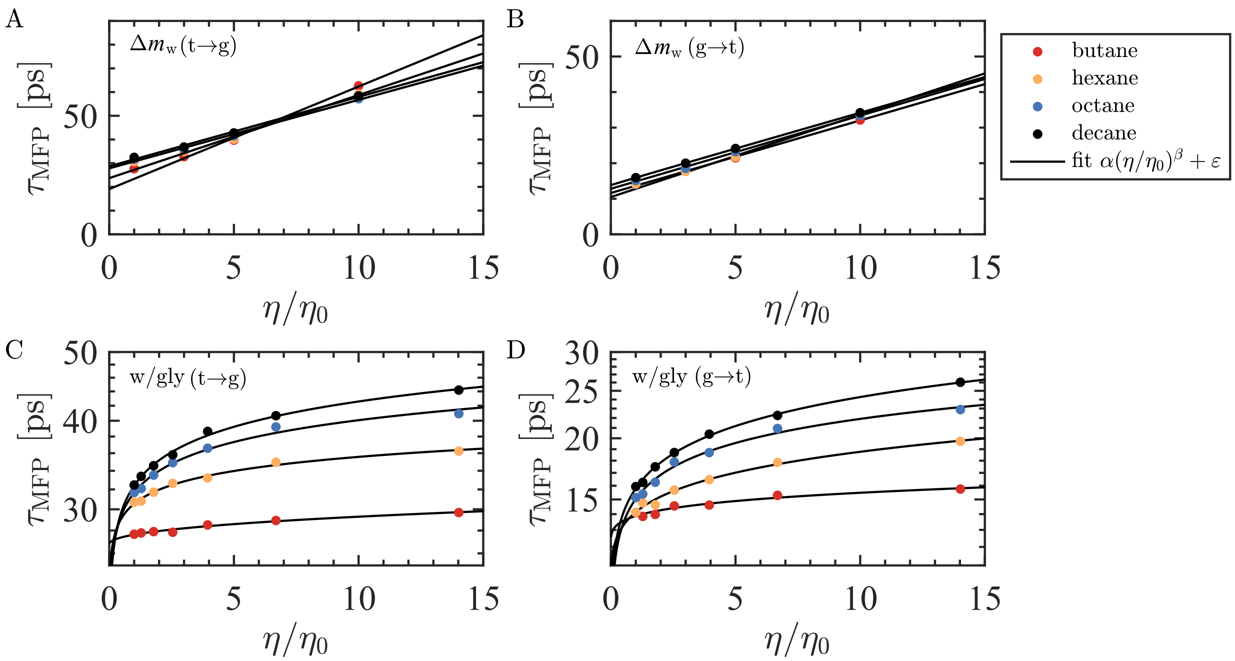

Supplementary Figure 10: Viscosity scaling of τ_{MFP} for the outer-most dihedral for the four alkanes. Coloured points are simulation data. The black solid lines are fits to $\tau_{MFP}(\eta/\eta_0) = \alpha(\eta/\eta_0)^\beta + \varepsilon$. (A) and (B) Super-heavy water trans→gauche and gauche→trans transitions, respectively. (C) and (D) Mixed water and glycerol solvent trans→gauche and gauche→trans transitions, respectively.

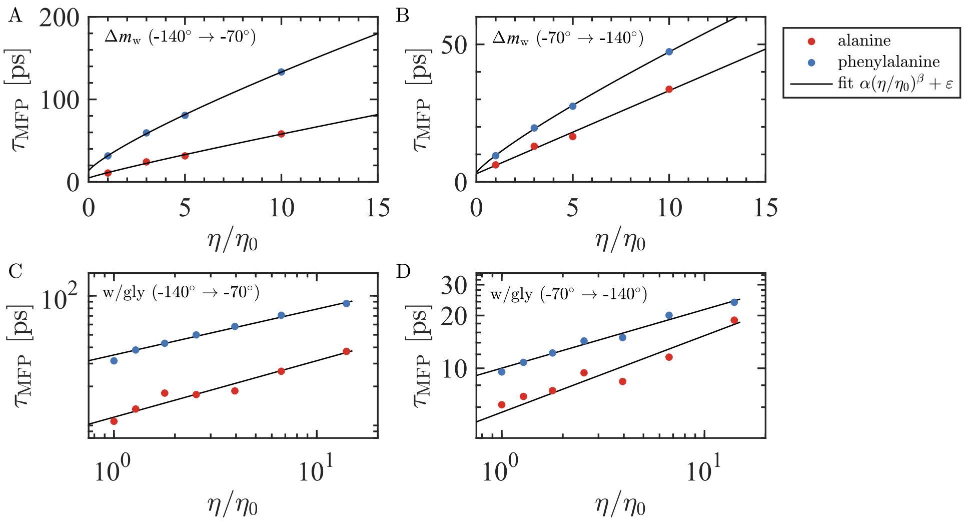

Supplementary Figure 11: Viscosity scaling of τ_{MFP} for the ϕ-dihedral for the capped alanine and phenylalanine amino acids. Coloured points are simulation data. The black lines are fits to $\tau_{MFP}(\eta/\eta_0) = \alpha(\eta/\eta_0)^\beta + \varepsilon$. (A) and (B) Super-heavy water $-140° \to -70°$ and $-70° \to -140°$ transitions, respectively. (C) and (D) Mixed water and glycerol solvent $-140° \to -70°$ and $-70° \to -140°$ transitions, respectively.

In Figs. Supplementary Figure 12 and Supplementary Figure 13, we show the full set of parameters obtained by fitting the viscosity scaling for the inner-most alkane dihedrals and the two capped amino-acids. The results for β and $\varepsilon/(\alpha+\varepsilon)$ are shown in the main manuscript.

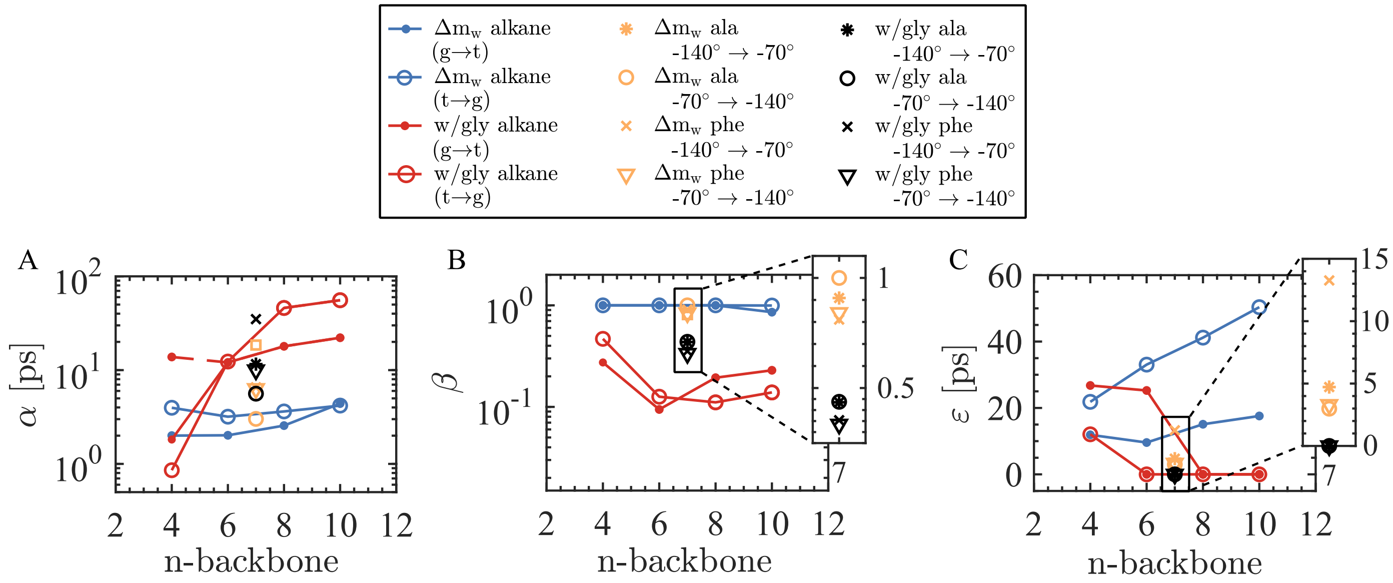

Supplementary Figure 12: Fitting parameters for the alkane inner-most dihedrals, and for the amino acid ϕ dihedrals. For the alkanes, the parameters are plotted as a function of the alkane chain length, where the carbon atoms are considered as the back-bone. For the amino acids, there is only a single backbone length. (A) The coefficient α as a function of backbone length. (B) The scaling exponential β as a function of backbone length. (C) The constant ε as a function of backbone length. The insets for (B) and (C) show the magnification for the amino acid values from the main panel.

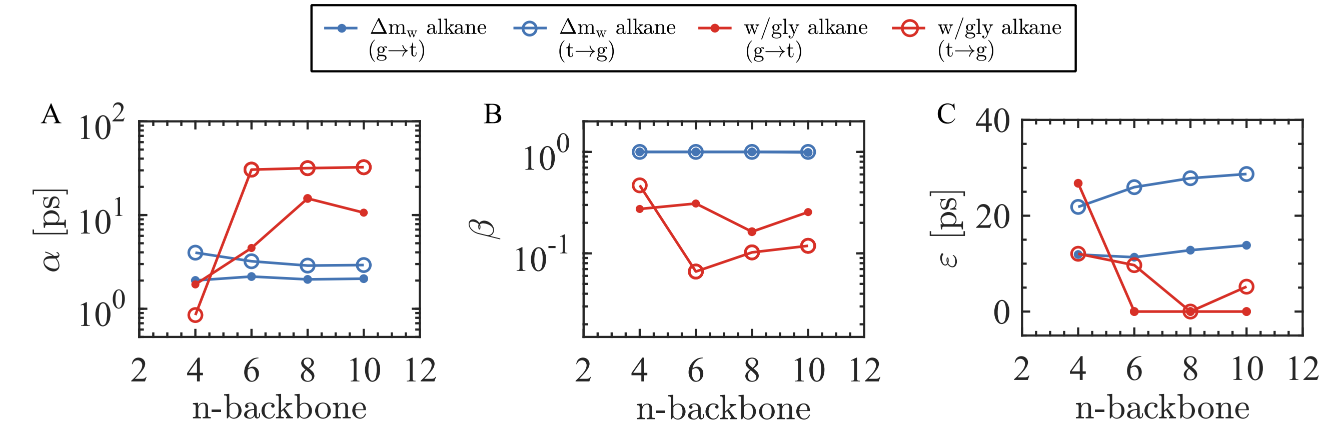

Supplementary Figure 13: Fitting parameters for the alkane outer-most dihedrals. (A) The coefficient α as a function of backbone length. (B) The scaling exponential β as a function of backbone length. (C) The constant ε as a function of backbone length.

Supplementary Note 10. Kramers' and Grote-Hynes predictions of butane and decane isomerization times

Since we directly extract the friction acting on the rotating dihedral, we can evaluate various reaction-rate theory predictions for the dihedral transition times and compare to the simulation results in Fig. 4A in the main manuscript. The Grote-Hynes prediction for barrier crossing with frequency-dependent friction is given by [16]

$$\tau_{\mathrm{GH}} = \frac{2\pi\omega_{\mathrm{max}}}{\lambda\omega_{\mathrm{min}}} \mathrm{e}^{\beta U_0}, \tag{S15}$$

where $\omega_{\mathrm{max}} = \sqrt{|U''_{\mathrm{max}}|/m}$ and $\omega_{\mathrm{min}} = \sqrt{U''_{\mathrm{min}}/m}$ are the barrier frequencies at the free energy maximum and minimum, with curvatures U''_{max} and U''_{min}. m is the effective mass of the dihedral, here taken to be constant, given by the equipartition theorem $m = k_{\mathrm{B}}T/\langle\dot{\theta}\rangle^2$. For the gauche→trans transition in butane, $|U''_{\mathrm{max}}| = 1.35\times10^{-3}\ k_{\mathrm{B}}T/\mathrm{deg}^2$ and $U''_{\mathrm{min}} = 6.25\times10^{-3}\ k_{\mathrm{B}}T/\mathrm{deg}^2$, with a barrier height $U_0 = 3.9\ k_{\mathrm{B}}T$. λ is the barrier reactive frequency determined by solving the Grote-Hynes equation

$$m\lambda^2 + \lambda\tilde{\Gamma}(\lambda) - m\omega_{\mathrm{max}}^2 = 0, \tag{S16}$$

where $\tilde{\Gamma}(\lambda)$ is the Laplace transform of the friction memory kernel, given by $\tilde{\Gamma}(\lambda) = \int_0^\infty \Gamma(t')\mathrm{e}^{-\lambda t'}dt'$, which we evaluate numerically. In the Markovian limit, i.e. when $\Gamma(t) = \gamma\delta(t)$, Eq. S15 reduces to the well-known Kramers prediction

$$\tau_{\mathrm{Kr}} = \left[\sqrt{\frac{\gamma^2}{4m^2} + \omega_{\mathrm{max}}^2} - \frac{\gamma}{2m}\right]^{-1} \frac{2\pi\omega_{\mathrm{max}}}{\omega_{\mathrm{min}}} \mathrm{e}^{\beta U_0}. \tag{S17}$$

In Supplementary Figure 14, we see that neither the Grote-Hynes, nor the Kramers theory, can consistently predict the viscosity scaling of τ_{MFP} for both butane and the decane inner dihedral, in both the super-heavy water and water-glycerol mixture. In particular, neither theory is able to predict the strongly disparate behaviour for the butane τ_{MFP}, as present in Fig. 2E of the main manuscript. It is not surprising that the Kramers prediction does not represent the simulation data well since it is only suitable for Markovian systems. The Grote-Hynes theory, however, explicitly incorporates non-Markovian effects. The breakdown of the Grote-Hynes theory in the small molecule regime indicates long-memory-time non-Markovian effects that the Grote-Hynes theory cannot capture.

Supplementary Table 2: Table of parameters relating to the free energy profiles of butane and decane. Parameters are used to calculate the Kramers predictions for τ_{MFP} that appears in Fig. 4A for the main article and in Supplementary Figure 14.

	RC	U''_{max}	U''_{min}	U_0	m
butane:	θ [deg]	-1.35×10^{-3} [$k_{\mathrm{B}}T/\mathrm{deg}^2$]	6.25×10^{-3} [$k_{\mathrm{B}}T/\mathrm{deg}^2$]	3.9 [$k_{\mathrm{B}}T$]	1.9×10^{-5} [unm^{2}deg^{-2}]
butane :	d_{14} [nm]	-3.22×10^{-3} [$k_{\mathrm{B}}T/\mathrm{nm}^2$]	2.05×10^{-3} [$k_{\mathrm{B}}T/\mathrm{nm}^2$]	3.11 [$k_{\mathrm{B}}T$]	11.4 [u]
decane:	θ [deg]	-1.35×10^{-3} [$k_{\mathrm{B}}T/\mathrm{deg}^2$]	6.25×10^{-3} [$k_{\mathrm{B}}T/\mathrm{deg}^2$]	3.9 [$k_{\mathrm{B}}T$]	1.9×10^{-5} [unm^{2}deg^{-2}]

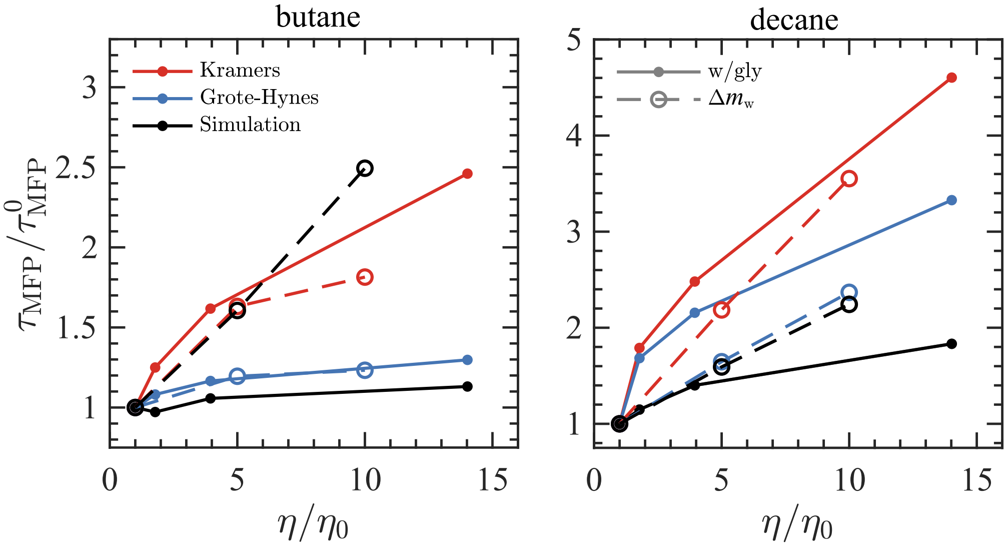

Supplementary Figure 14: Comparison of the Kramers prediction (Eq. S17) and the Grote-Hynes prediction (Eq. S15) to the simulation mean first-passage times τ_{MFP}, normalized by the neat water times τ^0_{MFP}, for the isomerization of butane and the inner dihedral of decane, plotted as a function of solvent viscosity.

Supplementary Note 11. Butane dihedral memory kernels and fitting parameters

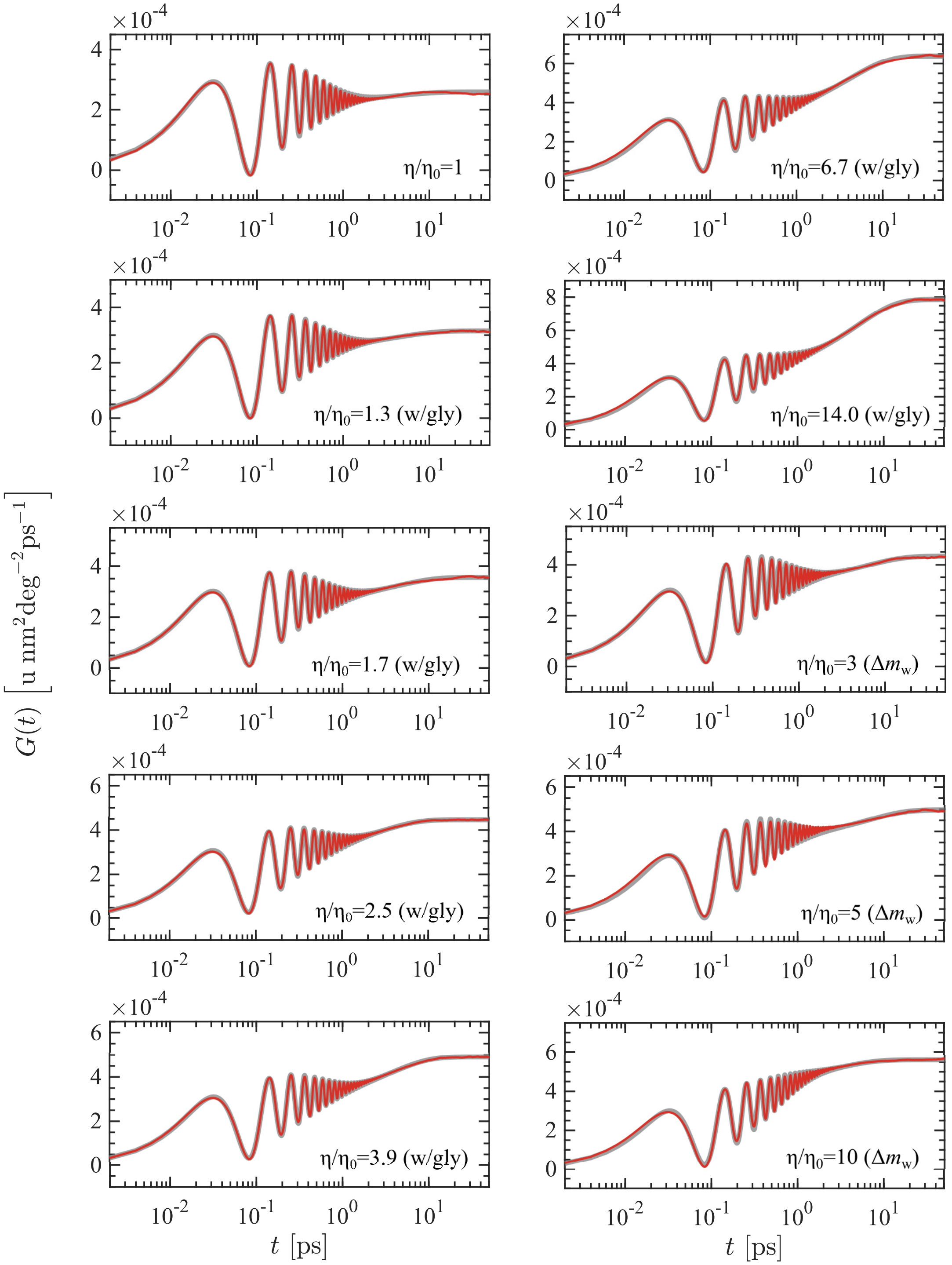

Supplementary Figure 15: The running integrals $G(t)$ associated with the memory kernels $\Gamma(t)$ for the butane dihedral for all solvent conditions. The red curves show $G(t)$ extracted from MD trajectories (see Supplementary Note 4). The thicker grey curves underlaid for each profile are the result for fitting Eq. S18 (Eq. 2 from the main text) to the MD results. Fitting is performed using non-linear least squares optimization.

In Supplementary Figure 15, we show the running integrals $G(t)$ of the memory kernels for the butane dihedral extracted from the MD trajectories under all solvent conditions. In Eq. 3 of the main manuscript, we propose a functional form for the the memory kernels $\Gamma(t)$ associated with the running integrals in Supplementary Figure 15. To fit to the extracted memory kernels, we fit the running integral form of the following series:

$$\Gamma(t) \approx \sum_{i=1}^{3} \frac{\gamma_i^{\mathrm{exp}}}{\tau_i^{\mathrm{exp}}} e^{-t/\tau_i^{\mathrm{exp}}} + \frac{(1+\omega_1\tau_1^{\mathrm{osc}})\gamma_1^{\mathrm{osc}}}{2\tau_1^{\mathrm{osc}}} e^{-t/\tau_1^{\mathrm{osc}}} \left[\cos(\omega_1 t) + \frac{\sin(\omega_1 t)}{\omega_1 \tau_1^{\mathrm{osc}}}\right], \tag{S18}$$

which is the same as Eq. 3 in the main text for $m = 3$ and $n = 1$. The resulting parameters from the 9-parameter fit in Eq. S18 are plotted in Figs. Supplementary Figure 16 and Supplementary Figure 17. These parameters are the same that appear in Fig. 4 in the main manuscript.

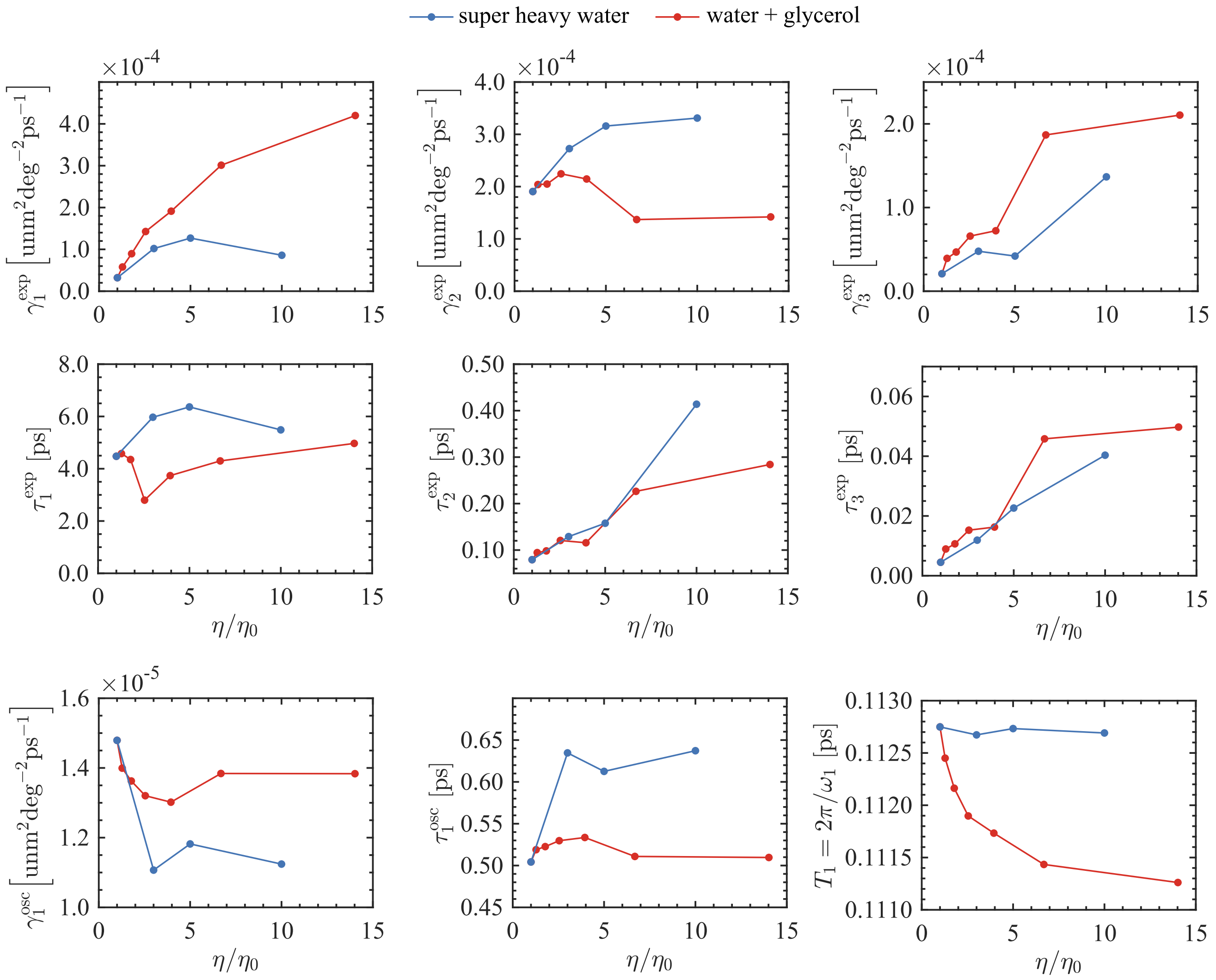

Supplementary Figure 16: Fitting parameters for memory kernels extracted from MD butane simulations for the dihedral angle reaction coordinate θ, plotted as a function of solvent viscosity. Comparison between super-heavy water solvent (blue) and aqueous-glycerol solvent (red). Fits are given by Eq. S18 and Eq. 2 in the main document, with m=3 and n=1, resulting in 9 fitting parameters for each kernel. γ_i^{exp} are the coefficients for the exponentially-decaying terms, and τ_i^{exp} are the time scales, for $i = 1, 2, 3$. γ_j^{osc} is the coefficient for the single decaying-oscillating term, T_1^{osc} is the period of oscillation ($T_1^{\mathrm{osc}} = 2\pi/\omega_1$, as presented in the main text), and τ_1^{osc} is the exponential time scale.

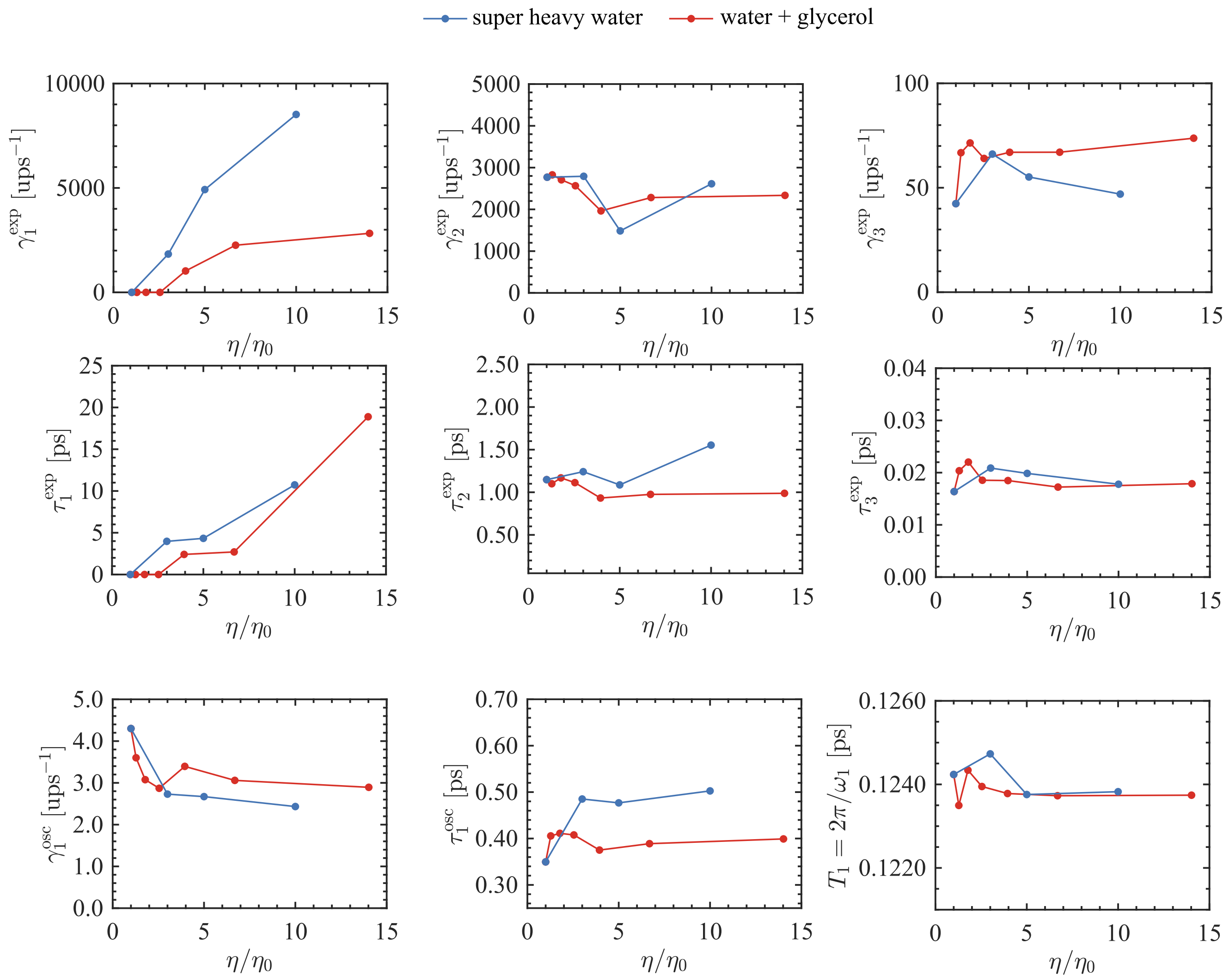

Supplementary Figure 17: Fitting parameters for memory kernels extracted from MD butane simulations for the intra-molecular distance reaction coordinate d_{14}, plotted as a function of solvent viscosity. Comparison between super-heavy water solvent (blue) and aqueous-glycerol solvent (red).

Supplementary Note 12. Effects of memory kernel oscillation on Grote-Hynes predictions

In Supplementary Figure 14, we present the Grote-Hynes predictions for the mean first-passage times of butane and decane isomerization using memory kernels extracted from simulations. As evident in Figs. 2A-D of the main manuscript and Figs. Supplementary Figure 3 and Supplementary Figure 15, the memory kernels extracted for the butane dihedral angle exhibit substantial contributions from oscillating modes. Our fitting results, obtained from Eq. 3 in the main text (and Eq. S18), indicate that only a single oscillating mode significantly contributes to the memory kernels. To evaluate the Grote-Hynes prediction, we numerically integrate the Laplace transform of the memory kernel to obtain the reactive frequency for the system, denoted as λ. To assess the influence of the oscillating mode on the Grote-Hynes prediction, we calculate τ_{GH} (Eq. S15) for the extracted MD kernels of the butane dihedral angle θ, the fully recombined fit of Eq. S18 (Eq. 3 from the main text) including all three exponential terms and the single oscillating term (fit all), a partially recombined version of Eq. S18, consisting solely of the three exponential components (fit exponential), and solely the single oscillating component (fit oscillating). We present the results in Supplementary Figure 18. The Grote-Hynes predictions, which incorporate the extracted kernels and the fully recombined fits, are in exact agreement. Upon removing the oscillating component, we observe a decrease in the magnitude of the predicted times, but no impact on the scaling. As a curious result, when we evaluate the predicted times associated with just the oscillating components, they are found to be equal for all systems. Consequently, we can conclude that the pronounced oscillations observed in the butane memory kernels influence the absolute times in the Grote-Hynes predictions while leaving the viscosity-scaling unaffected.

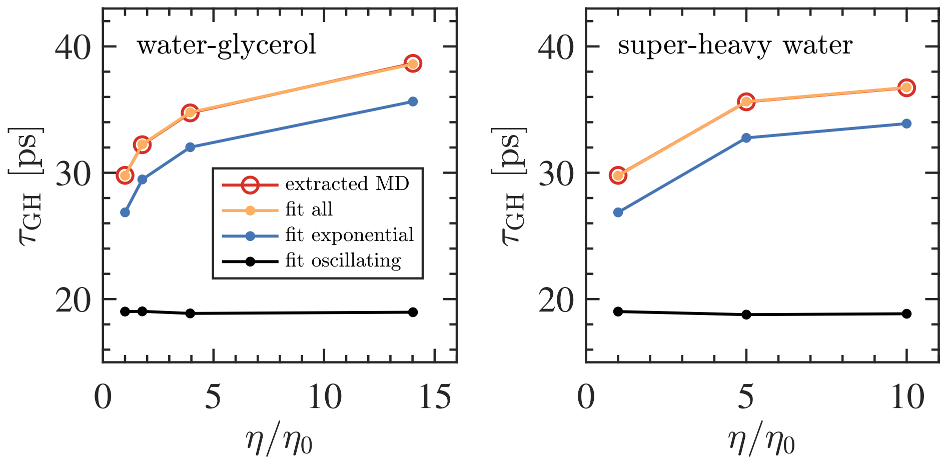

Supplementary Figure 18: Grote-Hynes predictions for butane isomerization times for systems with, and without, oscillating memory contributions, as measure in the dihedral angle reaction coordinate θ. The extracted MD results are the same as shown in the main manuscript (Fig. 4A). The memory kernels are fit with Eq. 3 from the main text (also Eq. S18 of the SI). Using the fitting results, we reconstruct the full memory kernel (fit all), only the exponential contributions (fit exponential), and only the single oscillating contribution (fit oscillating), and hence evaluate the Grote-Hynes prediction tau_{GH} for each case. Results are shown for the water-glycerol mixtures, and the super-heavy water solvents.

Supplementary Note 13. Water escape times as a solvent relaxation time scale

Here we show the escape times (τ_{esc}) for water as a representation of a solvent relaxation time scale and compare bulk relaxation (the escape time of water from a water-water pair interaction) to the relaxation of water around a butane molecule (the escape time of water from the neighbourhood of the CH_3 and CH_2 groups). The radial distribution functions ($g(r)$) for pairs of water oxygen atoms (O_w - O_w) and for interactions between water and the CH groups in the butane molecule are shown in Supplementary Figure 19A. The first and second peaks for the different $g(r)$ are used to define the escape transitions. The escape times shown in Supplementary Figure 19B are evaluated as the (all-to-all) mean first-passage times (see Supplementary Note 6) from the first hydration shell (first peak in the $g(r)$) to the second hydration shell (see [17] for details). Overall, we can see that all escape times are influenced by changes in viscosity, but that the increase in glycerol concentration has a smaller influence on the escape times than increasing the water mass.

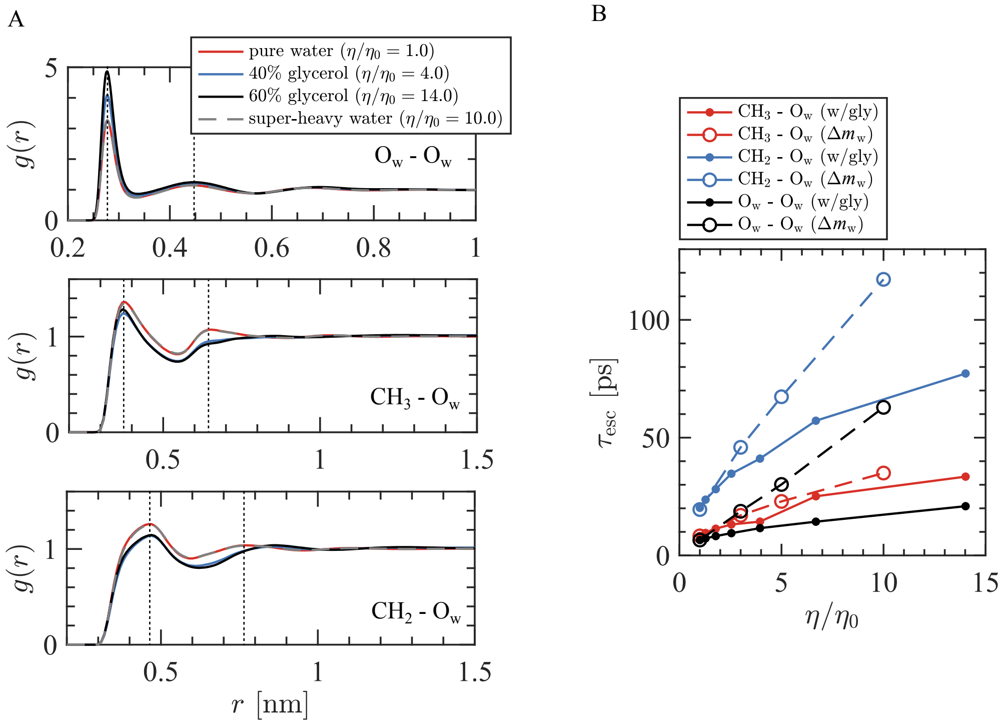

Supplementary Figure 19: (A) Radial distribution functions ($g(r)$) for the oxygen atoms in water pairs (O_w - O_w), as well as for the separations between the water oxygens and the different CH groups in the butane molecule (see Fig. 1B of the main manuscript). The $g(r)$ for pure water and super-heavy water η/η_0 are identical. The addition of glycerol changes the radial distribution function significantly. (B) Escape times τ_{esc} for water to pass from the first hydration shell to the second, as defined by the first and second peaks of the radial distribution functions (as indicated by the vertical broken lines).

Supplementary References

[1] J.-P. Ryckaert and A. Bellemans, Faraday Discussions of the Chemical Society **66**, 95 (1978).
[2] N.-S. Cheng, Industrial & Engineering Chemistry Research **47**, 3285 (2008).
[3] C. Ayaz, L. Scalfi, B. A. Dalton, and R. R. Netz, Physical Review E **105**, 54138 (2022).
[4] C. Ayaz, L. Tepper, F. N. Brünig, J. Kappler, J. O. Daldrop, and R. R. Netz, Proceedings of the National Academy of Sciences **118**, e2023856118 (2021).
[5] B. Kowalik, J. O. Daldrop, J. Kappler, J. C. F. Schulz, A. Schlaich, and R. R. Netz, Physical Review E **100**, 012126 (2019).
[6] J.-P. Ryckaert, G. Ciccotti, and H. J. C. Berendsen, Journal of Computational Physics **23**, 327 (1977).
[7] J. O. Daldrop, J. Kappler, F. N. Brünig, and R. R. Netz, Proceedings of the National Academy of Sciences **115**, 5169 (2018).
[8] R. O. Rosenberg, B. J. Berne, and D. Chandler, Chemical Physics Letters **75**, 162 (1980).
[9] D. W. Rebertus, B. J. Berne, and D. Chandler, The Journal of Chemical Physics **70**, 3395 (1979).

[10] H. S. Ashbaugh, S. Garde, G. Hummer, E. W. Kaler, and M. E. Paulaitis, Biophysical Journal **77**, 645 (1999).
[11] K. P. Travis and D. J. Searles, The Journal of Chemical Physics **125**, 164501 (2006).
[12] Q. Zhou, R. R. Netz, and B. A. Dalton, arXiv (2024), arXiv:2403.06604.
[13] S. Acharya and B. Bagchi, Physical Review E **107**, 24127 (2023).
[14] G. Barshtein, A. Almagor, S. Yedgar, and B. Gavish, Physical Review E **52**, 555 (1995).
[15] A. Sekhar, M. P. Latham, P. Vallurupalli, and L. E. Kay, The Journal of Physical Chemistry B **118**, 4546 (2014).
[16] R. F. Grote and J. T. Hynes, The Journal of Chemical Physics **73**, 2715 (1980).
[17] J. O. Daldrop, B. G. Kowalik, and R. R. Netz, Physical Review X **7**, 41065 (2017).
